# College Notes

**Published:** 1895-01

**Authors:** 


### COLLEGE NOTES.

The Veterinary Department of the Detroit College of Medicine has in contemplation the erection of a modern and commodious hospital. The course has not been lengthened, but the faculty has been strengthened by the addition of some two or three veterinarians. The second-year class numbers about twenty. The school suffered a serious loss in the death of Professor Reycraft last June.

The Indiana Veterinary College in its reorganization has greatly increased its corps of instructors. Several well-known veterinarians have been added to its teaching staff, which should greatly add to the efficiency of its work. The branches of theory and practice, anatomy, surgery, cattle pathology and contagious diseases, obstetrics and animal castration, canine pathology, meat and milk inspection, dentistry, hygiene, breeding and general management of domestic animals are all taught by veterinarians. A new college building is in course of erection.

The entrance requirements might be made higher, the curriculum increased, and a more fixed rule as to age for graduation, to the advantage of the profession. The college fees are very low.

The *Pacific Medical Journal* in its December issue bespeaks for the California Veterinary College a highly successful career. It has become affiliated with the California University. While most of the veterinary instructors are without experience in this line of work, still this is equally true of the teaching staff of every veterinary college at its inception in this country.

Prof. Leonard Pearson, of the Veterinary Department of the University of Pennsylvania, has been elected to the Chair of Veterinary Science at the State Agricultural College, Bellefonte, Pa.

As we go to press we learn of a change in the faculty of the California Veterinary College, the resignation of Dr. A. E. Buzard, professor of the principles and practice of equine medicine and dermatology, having been accepted, and the appointment of Dr. K. O. Steers, V.S., to the vacancy.

Veterinary Department of the Iowa Agricultural College matriculated twenty students for the class of '94 and '95. By an error of the printer in their catalogue the title of D.V.M. did not appear after the name of Dr. J. A. Replogle, one of the teaching staff, and this led us to fear that the number of veterinarians connected with the teaching staff had been decreased, an error we are very glad to correct.

